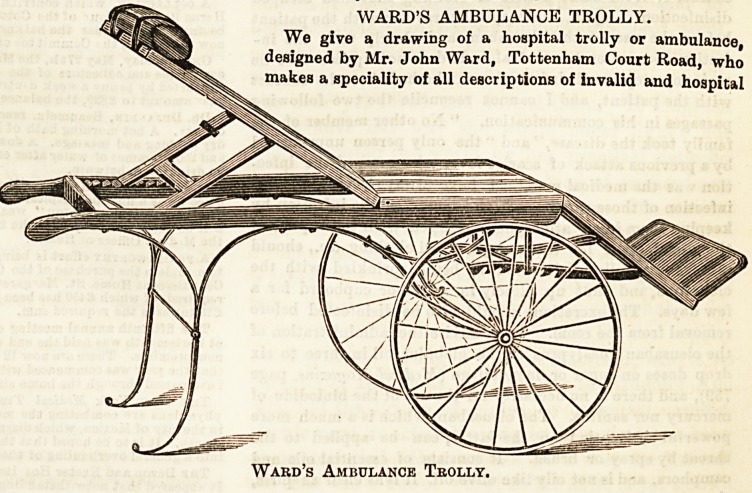# Ward's Ambulance Trolly

**Published:** 1893-06-24

**Authors:** 


					PRACTICAL DEPARTMENTS.
WARD'S AMBULANCE TROLLY.
We give a drawing of a hospital trolly or ambulance,
designed by Mr. John Ward, Tottenham Court Road, who
makes a speciality of all descriptions of invalid and hospital
furniture. The illustration explains itself, and shows at a
glance that the ambulance is light in make and well con-
structed. The rubber-tyred wheels makes the transport of
even heavy patients an easy matter, and are attended with
as little discomfort as possible to the injured person. Mr.
Ward is to be congratulated on the many improvements he
has introduced in designing and carrying out the construction
of these and other necessaries in hospital work.
WARD'S AMBULANCE TROLLY.
We give a drawing of a hospital trolly or ambulance,
designed by Mr. John Ward, Tottenham Court Road, who
makes a speciality of all descriptions of invalid and hospital
Ward's Ambulance Teolly.

				

## Figures and Tables

**Figure f1:**